# Characterisation of recombinant GH 3 β-glucosidase from β-glucan producing *Levilactobacillus brevis* TMW 1.2112

**DOI:** 10.1007/s10482-022-01751-7

**Published:** 2022-06-04

**Authors:** Julia A. Bockwoldt, Matthias A. Ehrmann

**Affiliations:** Chair of Microbiology, Technical University of Munich, Freising, Germany

**Keywords:** β-glucosidase, β-glucan, Exopolysaccharide, Glycoside hydrolase 3, Heterologous expression, *Levilactobacillus brevis*

## Abstract

*Levilactobacillus* (*L*.) *brevis* TMW 1.2112 is an isolate from wheat beer that produces *O2*-substituted (1,3)-β-D-glucan, a capsular exopolysaccharide (EPS) from activated sugar nucleotide precursors by use of a glycosyltransferase. Within the genome sequence of *L. brevis* TMW 1.2112 enzymes of the glycoside hydrolases families were identified. Glycoside hydrolases (GH) are carbohydrate-active enzymes, able to hydrolyse glycosidic bonds. The enzyme β-glucosidase BglB (AZI09_02170) was heterologous expressed in *Escherichia coli* BL21. BglB has a monomeric structure of 83.5 kDa and is a member of the glycoside hydrolase family 3 (GH 3) which strongly favoured substrates with β-glycosidic bonds. K_m_ was 0.22 mM for pNP β-D-glucopyranoside demonstrating a high affinity of the recombinant enzyme for the substrate. Enzymes able to degrade the (1,3)-β-D-glucan of *L. brevis* TMW 1.2112 have not yet been described. However, BglB showed only a low hydrolytic activity towards the EPS, which was measured by means of the D-glucose releases. Besides, characterised GH 3 β-glucosidases from various lactic acid bacteria (LAB) were phylogenetically analysed to identify connections in terms of enzymatic activity and β-glucan formation. This revealed that the family of GH 3 β-glucosidases of LABs comprises most likely exo-active enzymes which are not directly associated with the ability of these LAB to produce EPS.

## Introduction

The exopolysaccharide (EPS) formation by lactic acid bacteria (LAB) gained increased interest by the food industry in the past decades due to health-promoting effects and their application as natural viscosifier and thickening agents (Goh et al. 2005; Korcz et al. 2021; Moradi et al. 2021; Ruas-Madiedo et al. 2005; Zannini et al. 2016). The major advantages are the generally recognised as safe (GRAS) status of EPS forming LAB and further an in situ EPS enrichment of food products makes the use of additives (e.g., guar gum or pectin) redundant (Freitas et al. 2011; Velasco et al. 2009; Zannini et al. 2016). EPSs formed by LABs are either homopolysaccharides (HoPS) or heteropolysaccharides (HePS) (Badel et al. 2011; Fraunhofer et al. 2018b; Freitas et al. 2011; Notararigo et al. 2013). β-glucans (consisting solely of glucose monomers) are produced intracellularly by activated sugar nucleotide precursors and compared to HoPS have lower yields (Mozzi et al. 2006; Notararigo et al. 2013). Regarding the fermentation of foods, low yields and the degradation of in situ synthesized EPS are critical parameters for industrial applications (De Vuyst et al. 2001). Previous studies described the decrease of EPS concentrations with increasing fermentation periods of LAB either through enzymatic activity or physical parameters (Cerning et al. 1992; Degeest et al. 2002; Dierksen et al. 1995; Vuyst et al. 1998; Zannini et al. 2016). Degeest et al. (2002) and Pham et al. (2000) reported EPS degradation by cell extract of *Streptococcus thermophilus* LY03 and *Lacticaseibacillus rhamnosus* R. Glycohydrolases or glycoside hydrolases (GH), such as α-D-glucosidase, β-D-glucosidase, α-D-galactosidase or β-D-galactosidase, were found to be involved in EPS degradation, thus reducing the viscosity of the LAB culture broths. The GHs are grouped in more than 170 families which are classified based on their amino acid sequences. These enzyme families possess hydrolytic activities towards glycosidic bonds of carbohydrates and non-carbohydrate fractions. Furthermore, GHs can be classified into retaining and inverting enzymes depending on their catalytic mechanism. Inverting enzymes perform nucleophilic substitution and retaining enzymes form and hydrolyse covalent intermediates (Ardèvol et al. 2015; Koshland Jr. 1953; Naumoff 2011). The β-glucosidases of the GH 3 family, for example, retains the anomeric configuration of substrates and have a frequently occurring (β/α)_8_-barrel structure (Naumoff 2006; Rigden et al. 2003). GH 3 β-glucosidases could act as exo enzymes, able to hydrolyse terminal, non-reducing β-D-glycosyl residues including β-1,2-: β-1,3-; β-1,4-; β-1,6-linkages and/or aryl-β-glucosides with subsequent β-D-glucose release (Cournoyer et al. 2003; Harvey et al. 2000). It was demonstrated that in general the GH 3 family is one of the more abundant GH families in bacterial genomes. Moreover, the bacterial genome size correlated with the presence of this family, which means that smaller genomes (1066 ± 294 open reading frames (orf)) lacked the presence of GH 3 enzymes (Cournoyer et al. 2003).

*Levilactobacillus* (*L*.) *brevis* TMW 1.2112 is a wheat beer isolate which produces *O2*-substituted (1,3)-β-D-glucan, a HoPS. In this study, the genome sequence of *L*. *brevis* TMW 1.2112 was screened for GHs by in silico genome mining. One orf (AZI09_02170) was identified as putative β-glucosidase BglB (GH 3). BglB was heterologously expressed, characterised, and analysed for its ability to degrade isolated and purified β-glucan. Since β-glucosidases of LAB were previously described to be involved in EPS degradation (Degeest et al. 2002; Pham et al. 2000), BglB was of interest in this study also considering the β-linked EPS. Furthermore, the enzyme was compared with previously characterized lactic acid bacterial GH 3 β-glucosidases from the literature for a brief overview and to infer relations between the EPS forming and non-forming LAB.

## Material and methods

### Bacterial strains, plasmids, and cultivation

The EPS forming wheat beer isolate *L. brevis* TMW 1.2112 was cultivated in modified Man, Rogosa, and Sharpe medium (mMRS) with pH 6.2 at 30 °C as static cultures as previously described by (Fraunhofer et al. 2017; Schurr et al. 2013). *L. brevis* TMW 1.2112 and *Pediococcus claussenii* TMW 2.340 (isogenic with DSM 14800^ T^, and ATCC BAA-344^ T^) were cultivated in a modified semi-defined (SDM) at pH 5.5 with 20 g L^−1^ maltose as sole carbon source for EPS isolation. The isolation was performed according to Bockwoldt et al. (2021) except perchloric acid treatment (Dueñas-Chasco et al. 1997).

*Escherichia* (*E*.) *coli* BL21 (StrataGene®) cells and pBAD/*Myc*-His A (Invitrogen) were used for cloning and expression of the enzyme. Recombinant *E. coli* cells were grown in lysogeny broth (LB) Lennox medium (pH 7.2) at 37 °C with and 200 rpm or on solid LB medium with 1.5% (w/v) agar. Transformed cells were selected by adding 100 μg ampicillin mL^−1^ to the LB medium. The pBAD vector was constructed by introducing the appropriate DNA fragment of the β-glucosidase (AZI09_02170) into the NcoI and SalI sites of pBAD/myc-His by Gibson Assembly.

### Bioinformatic analysis

The previously sequenced genome of *L. brevis* TMW 1.2112 (Fraunhofer et al. 2018a) was used for similarity analysis of GH 3 by genome mining (Ziemert et al. 2016). The DNA and protein sequences were analysed by BLASTn and BLASTx, respectively (Altschul et al. 1990). Further characterizations of the enzymes and the GH family affiliation were performed by using CAZy (Lombard et al. 2014), functional information of the enzymes by UniProt (Consortium,  2020), and homology modelling was performed by SWISS-MODEL (Waterhouse et al. 2018). Prediction of a putative signal peptide was performed by using SignalP-5.0 (Armenteros et al. 2019).

### Construction of heterologous expression vector

The GH 3 β-1,3-glucosidase (AZI09_02170) gene was identified from the genome sequence of *L. brevis* TMW 1.2112 (GenBank accession No.: CP016797). The appropriate DNA sequence was amplified by PCR with Q5 High Fidelity DNA-Polymerase (NEB, Germany) using forward and reverse primers with pBAD overlaps 5′- CGTTTAAACTCAATGATGATGATGATGATGTTGGCGTAATAAGGTGTTTGCCCG-3′ and 5′- CGTTTTTTGGGCTAACAGGAGGAATTAACCATGGACATCGAACGAACGCTTGCTGAACTC-3′, respectively. Amplicons were generated by the PCR program as follows: initial denaturation at 98 °C for 30 s, followed by 30 cycles of 10 s at 98 °C, 20 s at 71 °C and 90 s at 72 °C with a final extension at 72 °C for 2 min. The PCR product was purified and integrated into the previously digested pBAD/*Myc*-His A vector by Gibson assembly (Gibson Assembly® Master Mix, NEB, Germany). The vector was digested using the enzymes *NcoI* and *SalI* (NEB, Germany) which simultaneously excised the *Myc*-region. The recombinant plasmid pBAD_bGLU was transformed into *E. coli* BL21 by the heat-shock method (Froger et al. 2007).

### Expression and purification

Positive clones of *E. coli* BL21 carrying the vector pBAD_bGLU were screened and selected for enzyme expression. LB medium containing 100 μg ampicillin ml^−1^ was inoculated with *E. coli* pBAD_bGLU and incubated at 37 °C and 200 rpm until OD_600 nm_ ≈ 0.5. The cells were induced with 0.25% L-arabinose (v/v) overnight at 15 °C and 200 rpm. In the next step, the cells were harvested by centrifugation at 3,000 × g for 10 min at 4 °C and resuspended in lysis buffer: 50 mM sodium phosphate, 300 mM NaCl, 5% glycerol, 1 mM phenylmethylsulphonyl fluoride (PMSF), 10 mM β-mercaptoethanol, pH 7.5. Cell disruption was performed using glass beads (Ø 2.85–3.45 mm) and a benchtop homogenizer (FastPrep®-24 MP, MP Biomedical Inc, Germany) in three cycles each 30 s. The cell debris was harvested by centrifugation 17,000 × g for 30 min at 4 °C and discarded. The supernatant including the his-tagged recombinant protein was added to nickel-nitrilotriacetic acid (Ni–NTA) crosslinked agarose resins (SERVA Electrophoresis GmbH, Germany) and purified according to the manufacturer’s protocol. The purified fractions were analysed and visualised on 12% sodium dodecyl sulphate–polyacrylamide gel electrophoresis (SDS-PAGE) gels by staining with Coomassie Brilliant dye Roti® Blue (Carl Roth GmbH + Co. KG, Germany). The protein concentration within the several fractions was determined by Coomassie (Bradford) protein assay kit using bovine serum albumin (BSA) as the standard (Thermo Fisher Scientific, Germany). Imidazole was removed by dialysis against 50 mM PBS buffer pH 6.8 overnight at 4 °C using 3.5 kDa dialysis tubing (SERVA Electrophoresis GmbH, Germany).

### Screening for the substrate specificity

The substrate specificity of the recombinant BglB and the cell lysate of *E. coli* BL21 was analysed using six different *p*-nitrophenyl phosphate (pNP) substrates: pNP β-D-glucopyranoside (pNPβGlc), pNP α-L-fucopyranoside (pNPαFuc), pNP β-D-fucopyranoside (pNPβFuc), pNP α-D-galactopyranoside (pNPαGal), pNP β-D-galactopyranoside (pNPβGal), and pNP β-D-maltoside (Carl Roth GmbH + Co. KG, Germany, Santa Cruz Biotechnology, Inc., USA and Merck, Germany). The purified and dialysed enzyme solution was incubated in 100 μL of 50 mM PBS buffer (pH7) with 2 mM pNP substrate at 37 °C for 2 h using a microtiter plate reader at 405 nm (SPECTROstar Nano, BMG Labtech GmbH, Germany). Determinations were done using biological duplicates each with at least technical duplicates.

In addition, API® ZYM (bioMérieux, Marcy-l´Étoile, France) test stripes were used for enzyme characterisation of the cell lysate samples from *E. coli* BL21 and induced *E. coli* pBAD_bGLU. The cell pellet of 5 mL culture volume was washed and resuspended with 2.5 mL PBS buffer (pH7). Cell disruption was done as previously described. The analysis was performed by inoculating each cupule of the test stripe with 65 μL of cell lysate and subsequently incubated for 4 h at 37 °C (Gulshan et al. 1990; Martínez et al. 2016). Water was added into the plastic trays to creating a humid atmosphere, preventing the enzymes from drying out. The reaction was terminated according to the manufacturer’s protocol. Colour changes were read after 5 min using a range from 0 to 5. While 0 represented no changes in the colour (0 nM substrate hydrolysed), represented a 5 a clear and strong colour change (≥ 40 nM substrate hydrolysed) and therefore a positive enzyme reaction (Baldrian et al. 2011). Determinations were done using biological duplicates.

### Influence of temperature and pH on β-glucosidase activity and stability

The optimal pH range of the recombinant BglB was measured at 37 °C in 50 mM PBS buffer containing 2 mM pNPβGlc with pH values ranging between 4 to 11 for 20 min. The temperature optimum was determined using 50 mM PBS buffer containing 2 mM pNPβGlc with the optimal pH incubated for 20 min at temperatures between 10 and 60 °C. The pH stability of the enzyme was determined in 50 mM PBS buffer with pH 4 to pH 11 for 2 h at 37 °C. The effect of the temperature on enzyme stability was tested by incubating the enzyme in 50 mM PBS (pH 7) for 2 h at various temperatures from 10 to 60 °C. The relative activities were calculated by released pNP from 2 mM pNPβGlc measured at 405 nm with a microtiter plate reader. Determinations were done using biological duplicates.

### Kinetic parameters of β-glucosidase

The Michaelis Menten constants (K_M_) and maximum reaction rate (V_max_) of the enzyme were determined in 50 mM PBS buffer (pH 7) at 37 °C using pNPβGlc concentrations between 0.01 and 20 mM (Johnson et al. 2011). An increase in absorbance by released *p*-nitrophenol was recorded at 405 nm with a microtiter plate reader. The recorded absorbance values of the first 4 min directly after adding the enzyme to buffers containing different pNPβGlc concentrations were used for the claculations. The kinetic constants of the β-glucosidase were calculated using Lineweaver–Burk plots (Lineweaver et al. 1934). Determinations were done using biological duplicates.

### Hydrolytic activity against isolated β-glucans

Isolated and purified bacterial β-glucan of *L. brevis* TMW 1.2112 and *P. claussenii* TMW 2.340 and curdlan (Megazyme Ltd., Ireland) were dissolved in 50 mM PBS buffer (pH 7) with a final concentration of 1 mg β-glucan mL^−1^. The β-glucan samples were inoculated with the recombinant β-glucosidase and incubated at 37 °C for 4 h. In addition, negative controls of the dissolved β-glucans were incubated without enzyme addition. Released D-glucose was enzymatically determined by glucose oxidase/peroxidase assay (GOPOD, Megazyme Ltd., Ireland) according to the manufacturer’s protocol, except adjustments of sample and reagent volumes. The assay was adapted to microtiter plate volumes with 50 μL sample volume and 150 μL of the GOPOD reagent. A standard curve using D-glucose was used to determine hydrolytic enzyme activity. Determinations were done using biological triplicates.

### Neighbour-joining tree of characterized GH 3 β-glucosidases of LAB

The visualization of the relationship of the GH 3 β-glucosidases was performed by reconstruction a phylogenetic tree.). A phylogenetic tree-based similarity matrix of amino acid sequences was constructed by the neighbour-joining method (Saitou and Nei 1987) using the Bionumerics^R^ software package V7.62 (Applied Maths, Belgium). Bootstrapping analysis was undertaken to test the statistical reliability of the topology of the tree using 1000 bootstrap resampling of the data.

## Results and discussion

### In silico* characterization of L. brevis TMW 1.2112 glycoside hydrolases*

Several glycoside hydrolases were identified within the genome sequence of *L. brevis* TMW 1.2112. The bioinformatic analyses revealed i.a. the GH 3 a β-glucosidase (bglB), GH 30 a glycosylceramidase, GH 65 a maltose phosphorylase and GH 88 a d-4,5 unsaturated β-glucuronyl hydrolase. The enzymatic activity of the β-glucosidase (BglB) was characterised. In addition, the putative hydrolytic activity towards bacterial β-glucan was tested.

### Characterisation of the GH 3 β-glucosidase gene and its ubiquity in other Lactobacillus strains

The BglB encoding gene AZI09_02170 (GenBank accession No.: ARN89439) of the beer spoiling and β-glucan forming *L. brevis* TMW 1.2112 which consists of 2256 bp was annotated as a putative intracellular glycoside hydrolase. The hydrolase with homology to the glycoside hydrolase family 3 encodes 751 amino acids with a molecular mass of 83.5 kDa. Sequence analysis with the BLAST program resulted similarities to several *L. brevis* glycoside hydrolases e.g., *L. brevis* ZLB004 (GenBank accession No.: AWP47268) with a 98% identity, a β-glucosidase-related glycosidase of *L. brevis* ATCC 367 (GenBank accession No.: ABJ65020) with 96% identity and two described thermostable β-glucosidases of *L. brevis* LH8 Bgy1 (GenBank accession No.: BAN07577) and Bgy2 (GenBank accession No.: BAN05876) isolated from Kimchi with 96% similarity. The thermostable β-glucosidases were analysed for the ability to form compound K from ginsenosides (Quan et al. 2008; Zhong et al. 2016a, 2016b). Michlmayr et al. (2010a) described a β-glucosidase of *L. brevis* SK3 isolated from a starter culture preparation for malolactic fermentation related to aroma compounds formation. Further sequence analysis resulted in a 67% identity with a thermostable β-glucosidase B (GenBank accession No.: VDC15331) of the (1,3)-β-D-glucan producing strain *Oenococcus* (*O.*) *oeni* IOEB 0205 (UBOCC-A-315001) (Ciezack et al. 2010; Dols-Lafargue et al. 2008; Gagné et al. 2011).

**Fig. 1:**
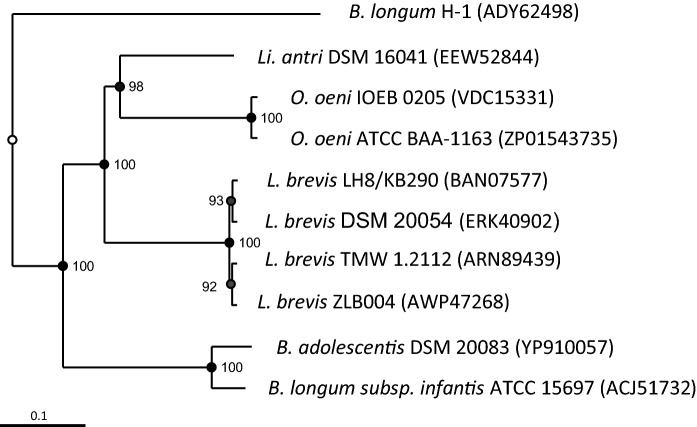
Neighbour-joining tree of characterised GH 3 β-glucosidases of LAB. Amino acid sequences of *L. brevis* TMW 1.2112 (ARN89439), *L. brevis* LH8 (KB290) Bgy1 (BAN07577.1), *L. brevis* ZLB004 (AWP47268), *L. brevis* DSM 20,054 (ATCC 14,869) (ERK40902), *Li. antri* DSM 16,041 (EEW52844), *O. oeni* IOEB 0205 (VDC15331), *O. oeni* ATCC BAA-1163 (ZP_01543735), *B. adolescentis* DSM 20,083 (ATCC 15,703) (YP_910057), and *B. longum subsp. infantis* ATCC 15,697 (ACJ51732) were used for alignment and phylogenetic analysis with Bionumerics V7.6.2. Bootstrap values above 50% are shown on each node and were calculated from 1000 replications. *B. longum* H-1 (ADY62498) is used as an outgroup. The bar indicates 1% sequence divergence

Phylogenetic analysis of the GH 3 β-glucosidases from LAB using *Bifidobacterium* (*B*.) *longum* H-1 as an outgroup resulted in four distinct groups: Bifidobacteria, *L. brevis* strains, *O. oeni* strains, and *Limosilactobacillus* (*Li*.) antri DSM 16,041 (Fig. 1). *O. oeni* IOEB 0205 and *O. oeni* ATCC BAA-1163 were both isolated from fermented wine and while *O. oeni* IOEB 0205 dispose of the glycosyltransferase family 2 gene (*gtf2*) resulting in β-glucan formation was *O. oeni* ATCC BAA-1163 lacking this gene (Ciezack et al. 2010). The *L. brevis* strains were isolated from faeces (ATCC 14,869 = DSM 20,054 and ZLB004), spoiled wheat beer (TMW 1.2112) and kimchi (LH 8). Though only *L. brevis* TMW 1.2112 carry the *gtf2* gene for β-glucan formation (Fraunhofer et al. 2018a; Michlmayr et al. 2015; Quan et al. 2008). The Bifidobacteria and *Li*. antri DSM 16,041 were isolated from gastrointestinal tract of humans and *gtf2* negative (Mattarelli et al. 2008; Reuter 1963; Roos et al. 2005). The phylogenetic analysis revealed that GH 3 β-glucosidases appear in LAB of different origins not specifically related to EPS production ability of the strains. In past studies possible degradation of EPSs by glycoside hydrolases of LABs was observed as decreased EPS yields over fermentation and lowered viscosity e.g., by *Lacticaseibacillus rhamnosus* R (formerly *Lactobacillus rhamnosus* R (Zheng et al. 2020)) and *Streptococcus thermophilus* LY03 (Cerning et al. 1992; Degeest et al. 2002; Pham et al. 2000; Vuyst et al. 1998; Zannini et al. 2016). However, the lack of hydrolytic enzymes from EPS forming LABs associated with its degradation was also described (Badel et al. 2011; Patel et al. 2012).

### Expression and purification of recombinant β-glucosidase

**Fig. 2:**
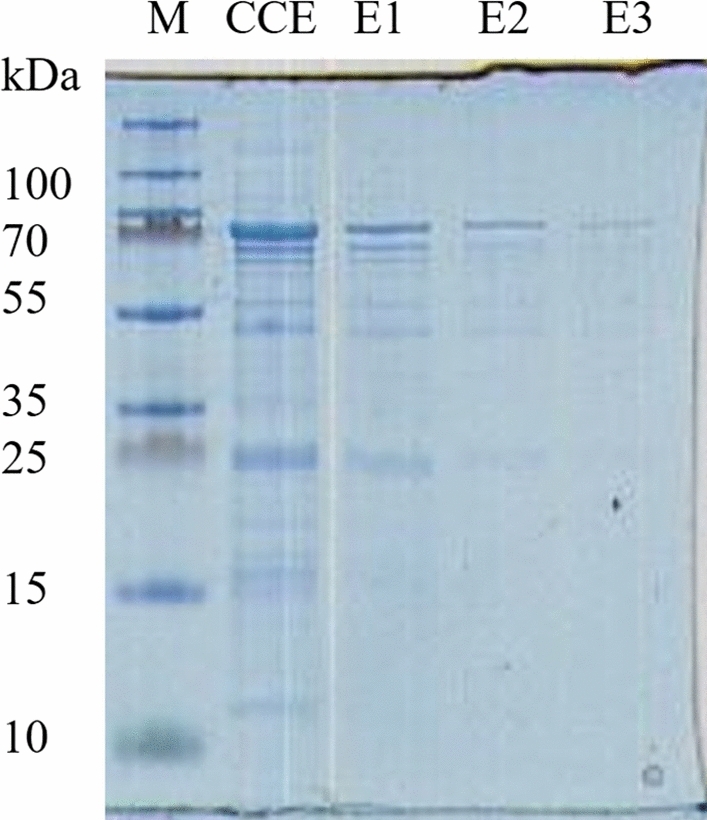
Coomassie brilliant blue-stained SDS-PAGE from crude cell-free extract (CCE) and purified protein fractions eluted from the Ni–NTA resins after three (E1–E3) rounds of purification; M, molecular mass marker (kDa), as indicated on the left

Within the sequence of *bgl*B no signal peptide sequence was predicted and only the stop codon was removed regarding Ni–NTA affinity purification via the poly-histidine tag coded within the expression vector. The sequence of *bgl*B was amplified by PCR and integrated into the expression vector pBAD/*Myc*-His and expressed in *E. coli* BL21. To maximize the protein yield, different inducing agent concentrations and inducing temperatures were tested and resulted an optimum concentration of 0.25% L-arabinose (v/v) at 15 °C overnight (García-Fraga et al. 2015; Sørensen et al. 2005). The intracellular formed enzyme was purified with Ni–NTA from the crude cell extract. The molecular mass of the enzyme was calculated via the amino acid sequence and resulted 83.5 kDa which corresponded with the bands of the elution fractions in SDS-PAGE gel stained with Coomassie (Fig. 2).

### Substrate spectrum

Seven different pNP substrates were used analysing the specific enzyme activity at 37 °C within 2 h with a microtiter plate reader (Table 1). The results for BglB indicated specificities for β-D-linked glycosides. Furthermore, the cell lysate of untransformed *E. coli* Bl21 was tested for enzymatic activity using the pNP substrates, which was negative i.a. for pNPβGlc. A significantly higher specificity of BglB was observed with pNPβGlc compared to the other substrates tested. This was confirmed by API® ZYM analyses resulting a strong colour change (≥ 40 nM substrate hydrolysed) and subsequently a positive enzyme reaction. However, a difference was observed for the β-galactosidase activity which was negative with the API® test and positive using pNPβGal. This might be associated with the different substrates type used in both analyse. The specificity of β-glucosidases for pNPβGlc is well described in several studies of different bacterial hosts (Chen et al. 2017; Fusco et al. 2018; Méndez-Líter et al. 2017; Michlmayr et al. 2010a, 2010b; Zhong et al. 2016a). Due to the high affinity of the enzyme to pNPβGlc this substrate was used in the following analysis.

**Table 1:** Substrate specificity of the GH 3 β-glucosidase from *L. brevis* TMW 1.2112. Values are means of biological duplicates including standard deviations

Enzymatic activity	Substrate	Hydrolytic activity	Relative activity [%]
β-glucosidase	pNPβGlc	+	100 ± 0.0
α-fucosidase	pNP α-L-fucopyranoside	−	0
β-fucosidase	pNP β-D-fucopyranoside	+	2.5 ± 0.2
α galactosidase	pNP α-D-galactopyranoside	−	0
β-galactosidase	pNP β-D-galactopyranoside	+ ^a^	1.4 ± 0.0
β-maltosidase	pNP β-D-maltoside	−	0

API® ZYM reaction			Activity 0–5
Phosphatase alkaline	2-naphthyl phosphate	+ / − ^a^	1
Esterase lipase (C 8)	2-naphthyl caprylate	−	0
Lipase (C 14)	2-naphthyl myristate	−	0
Leucine amino-peptidase	L-leucyl-2-naphthylamide	+ / − ^a^	1
Valine amino-peptidase	L-valyl-2-naphthylamide	+ / − ^a^	1
Cystine amino-peptidase	L-cystyl-2-naphthylamide	−	0
Trypsin	N-benzoyl-DL-arginine-2-naphthylamide	−	0
Chymo-trypsin	N-glutamine-phenylalanine-2-naphthylamide	−	0
Phosphatase acid	2-naphthyl phosphate	+ / − ^a^	4
Phospho-amidase		+ / − ^a^	2
α-galactosidase	6-Br-2-naphthyl-α-D-galactopyranoside	−	0
β-galactosidase	2-naphthyl-β-D-galactopyranoside	−	0
β-glucuronidase	Naphthol-AS-Bl-β-D-glucuronide	+ ^a^	2
β-glucosidase	6-Br-2-naphthyl-β-D-glucopyranoside	+	5
β-glucosaminidase	1-naphthyl-N-acetyl-β-D-glucosaminide	−	0
α-mannosidase	6-Br-2-naphthyl-α-D-mannopyranoside	−	0
α-fucosidase	2-naphthyl-α-L-fucopyranoside	−	0

^a^Was additionally positive for *E. coli* BL21

### Effects of temperature and pH on the enzyme activity and stability

**Fig. 3:**
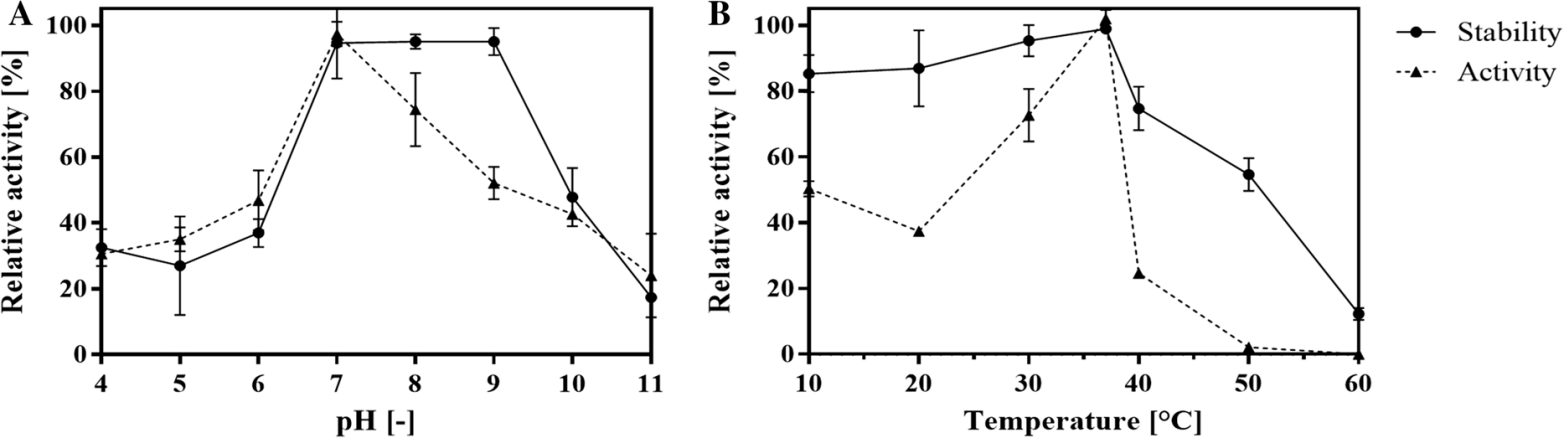
**A** Effects of pH changes, **B** effects of temperature changes on enzyme stability and activity of the recombinant β-glucosidase. Values are means of triplicates including standard deviations

The pH stability (Fig. 3A) of the recombinant β-glucosidase was analysed at a range of pH 4–11 and resulted in a hight stability at pH values between 7 and 9 with ≥ 95% relative activities. Under acidic conditions (pH 4–6) the enzyme stability decreased and was < 40%, the stability values at pH 10 and 11 were similar. The optimum pH for enzyme activity was observed at pH 7. Next to the pH conditions, the enzyme stability at different temperatures (10–60 °C) was determined and displayed the maximum at 37 °C (Fig. 3B). Between 10 and 37 °C the relative activity was ≥ 80% decreasing to 12% at 60 °C. The temperature optimum for enzymatic activity was measured at 37 °C, temperatures above or below resulted only ≤ 40% relative activity. The described β-glucosidases of *L. brevis* SK3 and *L. brevis* LH8 showed optimal activities at pH 5.5 and 45 °C and pH 6–7 and 30 °C, respectively. Furthermore, characteristics of described GH 3 β-glucosidases including *O. oeni* species, Bifidobacteria and other LAB were compared (Table 2). Revealing that the temperature optima of β-glucosidases from *L. brevis* strains were in average lower compared to thermostable β-glucosidases of Bifidobacteria or *O. oeni* strains. In general, the pH optima ranged between 4.5 and 7 and temperature optima between 30 and 55 °C (Michlmayr et al. 2010b, 2015; Zhong et al. 2016b).

**Table 2:** Properties of GH 3 β-glucosidases from lactic acid bacteria

Organism	K_m_ [mM]^a^	V_max_ [µM min^−1^]	k_cat_ [s^−1^]	pH optimum [−]	Temp. optimum [°C]	Substrate spectra	Reference
*L. brevis* TMW 1.2112 *	0.22	77	60	7	37	pNPβGlc, pNPβFuc, pNPβGal, (further substrates are listed in Table 1)	This study
*L. brevis* SK3	0.22	n.d	n.d	5.5	45	pNPβGlc, pNP-β-D-xylopyranoside (pNPβXyl), pNP-α-L-arabinopyranoside (pNPαAra)	(Michlmayr et al. 2010a)
*L. brevis* ATCC 14,869 = DSM 20,054*	0.63	47	66	4.5	45	Cellobiose, Salicin, pNPβGlc, pNPβXyl, pNPαAra, n-Octyl-β-D-glucopyranoside, Deoxynivalenol-3-O-β-D-glucopyranoside, Nivalenol-3-O-β-D-glucopyranoside, HT-2-toxin-3-O-β-D-glucopyranoside	(Michlmayr et al. 2015)
*L. brevis* LH 8 Bgy1*	n.d	n.d	n.d	6	30	oNPβGlc, pNPβGlc	(Zhong et al. 2016a)
*L. brevis* LH 8 Bgy2	n.d	n.d	n.d	7	30	oNPβGlc, pNPβGlc	(Zhong et al. 2016b)
*Limosilactobacillus antri* DSM 16,041	n.d	n.d	n.d	6	45	pNPβGlc	(Kim et al. 2017)
*Lacticaseibacillus casei ATCC 393*	16	n.d	n.d	6.3	35	pNPβGlc, pNPαGlc, oNPβGlc, pNPβGal, Methyl-β-D-glucoside, Salicin, Prunassin, Cellobiose,	(Coulon et al. 1998)
*O. oeni* IOEB 0205 (UBOCC-A-315001)*	n.d	n.d	n.d	n.d	n.d	pNPβGlc, pNPαGlc, pNPβXyl, pNPαAra, p-nitrophenyl-α-L-rhamnopyranoside	(Gagné et al. 2011)
*O. oeni* ATCC BAA-1163*	0.17	n.d	n.d	5.5	45–50	pNPβGlc, pNPβXyl	(Michlmayr et al. 2010b)
*O. oeni *ST81	0.38	0.00521	n.d	5.0	40	pNPβGlc	(Mesas et al. 2012)
*O. oeni* 31MBR	1.05	0.00096	n.d	4.5–5	45	pNPβGlc	(Dong et al. 2014)
*Bifidobacterium adolescentis* DSM 20,083 (ATCC 15,703)*	0.32	0.00037	88	6.5	45	pNPβGlc, pNPβXyl	(Florindo et al. 2018)
*Bifidobacterium adolescentis* DSM 20,083 (ATCC 15,703)*	1.1	68	94	5.5	55	pNPβGlc, pNPβXyl, pNPαAra, pNPβGal, Cellobiose, Salicin, Quercetin-3-O-β-D-glucopyranoside n-Octyl-β-D-glucopyranoside Deoxynivalenol-3-O-β-D-glucopyranoside Nivalenol-3-O-β-D-glucopyranoside HT-2-toxin-3-O-β-D-glucopyranoside	(Michlmayr et al. 2015)
*Bifidobacterium longum subsp. infantis* ATCC 15,697*	0.27	n.d	24	6	30	pNPβGlc, pNPβXyl, pNPαAra	(Matsumoto et al. 2015)
*Bifidobacterium longum* H-1	0.83	57	n.d	5.5	35–37	pNPβGlc, Ginsenoside Rb1, Loganin, Arctin, Arbutin	(Jung et al. 2012)

*Temp*. = temperature, *n.d.* not determined

^**⁕**^Sources included in Neighbour-joining tree of characterized GH 3 β-glucosidases

^a^K_m_ was analysed using pNPβGlc, as substrate

### Kinetic parameters

The kinetic parameters of BglB were calculated by Lineweaver–Burk plot using pNPβGlc as substrate at various concentrations. The enzyme had a high affinity for the substrate revealed by a low *K*_*m*_ which was 0.22 mM. The maximal rate (*V*_*max*_) was 77 μM · min^−1^, *k*_*cat*_ was 59.58 s^−1^ and the catalytic efficiency (*k*_*cat*_*/K*_*m*_) was 8.3 · 10^3^ s^−1^ mM^−1^. The *K*_*m*_ value of the β-glucosidase from *L. brevis* SK3 measured with pNPβGlc was 0.22 mM (Michlmayr et al. 2010a). Further *K*_*m*_ values of GH 3 β-glucosidase (Table 2) from LABs ranged between 0.17 mM and 16 mM using pNPβGlc as substrate (Coulon et al. 1998; Sestelo et al. 2004).

### Enzymatic hydrolysis of β-1,3-linked glucan by recombinant β-glucosidase

**Fig. 4:**
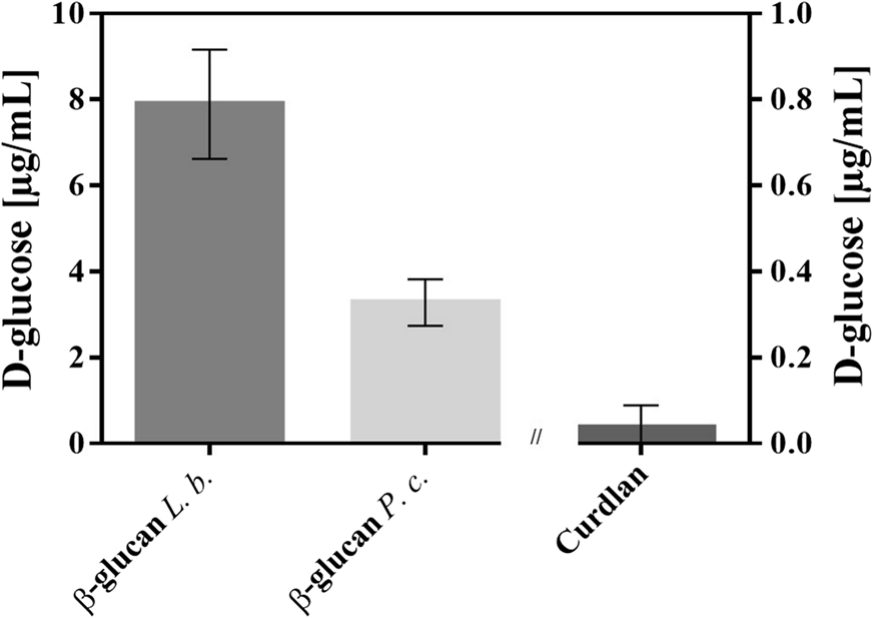
β-glucans of 3 different sources (*L. brevis* (L. b.), *P. claussenii* (P. c.) and curdlan) were incubated with the recombinant β-glucosidase for 4 h at 37 °C, released D-glucose concentrations based on enzymatic determination. Values are means of triplicates including standard deviations

The motivation of this study was to characterise the carbohydrate active enzyme BglB of the β-1,3-linked glucan producing LAB *L. brevis* TMW 1.2112. The involvement of BglB in the degradation of cell-own EPS was additionally ivestigated. Three β-glucan isolates including cell-own β-glucan of *L. brevis* TMW 1.2112 were incubated with the purified recombinant enzyme and released D-glucose was quantified (Fig. 4). Curdlan a linear β-1,3-linked glucan resulted a negligible amount of free D-glucose after incubation with the enzyme which was rather a result of dissolving than enzymatic activity. Furthermore, since curdlan is insoluble in water, this could affect the availability of the polymer for enzymatic degradation (Koumoto et al. 2004; Zhang et al. 2014). Released D-glucose from β-glucan produced by *L. brevis* TMW 1.2112 and *P. claussenii* TMW 2.340 were significantly higher, however the D-glucose concentration were still low with a maximum of ~ 8 μg D-glucose · mL^−1^ (*L. brevis* TMW 1.2112 β-glucan). The solubility of the isolated bacterial β-glucans was likewise low which could be caused by the extraction conditions, structure, and degree of polymerization (Bohn et al. 1995; Havrlentova et al. 2011; Virkki et al. 2005). Furthermore, the purification process in some cases affects the structure integrity due to harsh chemicals and physical methods as used in this study e.g. ethanol precipitation, benchtop homogenizer, and freeze drying with subsequent resuspending (Goh et al. 2005). In addition, *L. brevis* TMW 1.2112 and *P. claussenii* TMW 2.340 synthesize likewise high-molecular weight β-glucans similar to that of *P. parvulus* 2.6R and *O. oeni* IOEB 0205, with molecular mass of 3.4 · 10^4^ to 9.6 · 10^6^ Da and 8.0 · 10^4^ to ≥ 1 · 10^6^ Da, respectively. (Ciezack et al. 2010; Dols-Lafargue et al. 2008; Werning et al. 2014). High-molecular β-1,3-linked glucan are described as insoluble in water (Bohn et al., 1995). Moreover, the degradation of β-glucan is more likely performed by more than one hydrolytic enzyme, especially as the characterized β-glucosidase (AZI09_02170) is an intracellularly expressed enzyme of *L. brevis* TMW 1.2112. Furthermore, in our previous study, we showed that the decrease in viscosity of *L. brevis* TMW 1.2112 culture broth could not be explained by the degradation of late expressed enzymes including BglB. However, the viscosity decrease indicated the degradation of high-molecular β-glucan which may have been caused by so far unknown enzymes of this strain (Bockwoldt et al. 2022).

According to the finding of this study and by the comparison of the GH 3 β-glucosidases from other LAB, BglB seemed to be an exo-active enzyme able to hydrolyse terminal, non-reducing β-D-glycosyl residues of substrates. This restricted hydrolytic activity could be an explanation of the low released D-glucose amounts from β-glucan. Moreover, the β-glucosidase is most likely active on smaller carbohydrates and not high-molecular weight β-glucan. However, it might be involved to a later stage in polymer degradation e.g., after digestion with an endo-glucanase or if (partial) cell lysis occurs (Degeest et al. 2002; Pham et al. 2000). In preliminary experiments endo- and exo-glucanases of different origin (*Trichoderma* sp., and *Aspergillus oryzae*) further including a β-glucosidase from *Aspergillus niger* were used for the hydrolysis of the isolated bacterial β-glucan. Among others the GEM-assay (Danielson et al. 2010) was performed and resulted similar low D-glucose amounts after enzymatic digestion (data not shown) which again could be associated to the hurdles of β-glucan purification and resuspension.

In conclusion, we have identified and characterised the β-glucosidase BglB of the beer spoiling and β-glucan forming *L. brevis* TMW 1.2112 with a molecular mass of 83.5 kDa which strongly favoured substrates with β-glycosidic bonds and is apparently an exo-active enzyme. Even though the start of β-glucan degradation was observed and might be in greater extent after a longer incubation period, the in vivo identification of involved enzymes in bacterial β-glucan degradation e.g., by proteomic analysis is more favourable. Thus, the weak solubility of isolated β-glucan and feasible structural changes are eliminated and analysis of the enzymes activity under native conditions is enabled. However, it also looks like, given the phylogenetic analysis and characterization of GH 3 β-glucosidases from LABs, that this very enzyme family is not explicitly relevant to the EPS degradation.

## Data Availability

Data sharing not applicable.
